# Experiences of Older Aboriginal People in Navigating Transport Systems in an Urban Setting: An Indigenous Perspective on Transport Access, a Social Determinant of Health

**DOI:** 10.3390/ijerph192113778

**Published:** 2022-10-23

**Authors:** Tracey Ma, Rebecca Ivers, John Solar, Aaron Simon, Evelyne de Leeuw, Kathleen Clapham

**Affiliations:** 1 School of Population Health, UNSW Sydney, Sydney, NSW 2052, Australia; 2 Healthy Urban Environments Collaboratory, Maridulu Budyari Gumal SPHERE, Liverpool, NSW 2170, Australia; 3 Ngarruwan Ngadju First Peoples Health and Wellbeing Research Centre, University of Wollongong, Wollongong, NSW 2522, Australia

**Keywords:** mobility, access, age-friendly transportation, social inclusion, quality of life

## Abstract

Background: In Australia, Aboriginal people are underserved by the transport system and are less able to easily get to places they need to go than others. This is a part of a larger pattern of exclusion and inequity for Aboriginal people which affects their health, wellbeing, and social participation. Guided by a decolonising framework, this research explored how older Aboriginal people, whose pivotal roles in their families and communities require their mobility, experience the transportation system, providing an Indigenous-centred view of the accessibility of transportation options in society. Methods: Interviews drawing from the yarning technique were conducted with ten older Aboriginal people living in Greater Western Sydney and analysed qualitatively. Results: In addition to the cognitive labour required to decipher the rules of the transport system and organise commitments to match the scheduling of transport services, older Aboriginal people in this study experienced stigmatising attitudes and condescending treatment from service professionals and the public when traveling. Conclusions: This study suggests three potential ways that the current trajectory that underserves older Aboriginal people could be disrupted, relating to service design, the diversity and inclusion agenda, and the social determinants of Indigenous health.

## 1. Introduction

Mobility is a basic human need and a universal human right [1]. As mobility is achieved through transportation, it is imperative that transportation options in society are accessible to the individuals that live within it. However, disparities exist in people’s access to transportation, resulting in some individuals being unable to travel when and where needed without difficulty. In Australia, this ‘transport disadvantage’ is often discussed in relation to specific spatial areas [2,3,4,5,6], such as outer city suburbs [7]. Receiving less attention is Indigenous peoples’ experience with and access to transport [8].

The Australian Indigenous population, the Aboriginal and Torres Strait Islander peoples, is a broad heterogeneous group within which exists enormous cultural, social, linguistic, and geographical diversity [9]. The vast majority (79%) of Aboriginal and Torres Strait Islander people live in urban areas [10]. Aboriginal people have culturally specific needs and aspirations for access, mobility, and social participation [11,12,13,14], which ‘do not fit neatly into’ the mainstream transport system [14]. The failure of transport systems to address these needs means that Aboriginal people are underserved by the transport system. Furthermore, as a result of this diminished access to transport, Aboriginal people are less able to easily get to places that they need to go compared to the rest of the population [15].

This reduced mobility impedes Aboriginal people’s health, wellbeing, and social participation. Specifically, it impedes their capacity to access health care and other services; pursue social and economic opportunities; maintain kinship obligations, cultural priorities, and responsibilities to ancestral lands; and function autonomously within the parameters of the law [11,13,14,15,16]. Importantly, these implications do not only impact the lives of Aboriginal people, as individuals, but rather touch entire communities. This may especially be so when older Aboriginal people are unable to travel when and where needed without difficulty, as they play pivotal roles in maintaining the wellbeing of their families and communities and have important cultural responsibilities to fulfill that require their mobility, such as volunteering at schools or attending committee meetings [9].

The underserving of Aboriginal people’s specific transport needs stem from the ongoing legacies of colonisation, which have created patterns of exclusion and inequity for Aboriginal people [17,18] and significantly undermined their well-being [19]. While the social determinants of Indigenous health and Indigenous rights have been acknowledged in policy rhetoric, they are absent from policy implementation [20]. The lack of policy action attending to the needs and interests of Aboriginal people has been attributed to a policy discourse that frames the underserving of Aboriginal people by mainstream services as a failure of Aboriginal people to fit in with dominant norms and institutions [20]. This discourse sees Aboriginal people as ‘Other’, dismissing their perspectives, experiences, and values.

Rather than accept conventional discourse that does not adequately attend to the experiences of Aboriginal people, a decolonising approach, instead, questions the legitimacy of ideas and beliefs that maintain the subjugation of Aboriginal people and, in doing so, privileges the wisdom of Aboriginal people [21,22]. With that intent, this study explores how Aboriginal people, specifically older Aboriginal people, experience the transportation system in order to provide an Indigenous-centred view of the accessibility of transportation options in society.

## 2. Methods

This qualitative research was conducted within a decolonising framework. A decolonising framework privileges the Indigenous presence and situates the research within larger historical, political, and cultural contexts [23,24,25,26]. This research prioritises Aboriginal people’s perspectives, experiences, and values; its scope, processes, and materials were established with input from the Aboriginal community; and it was carried out under the leadership of a senior Aboriginal researcher (KC). By focusing on people’s lived experiences in a given place, this research also takes inspiration from phenomenology as a place-based methodology of inquiry [27]. Guided by the phenomenological tradition, the experience of individuals is the unit of analysis [28,29]. This research followed the Australian guidelines for ethical research with Aboriginal and Torres Strait Islander people [30,31]. Ethical approval of this research was granted by the Aboriginal Health & Medical Research Council of New South Wales (1741/20).

### 2.1. Setting

**Figure 1:**
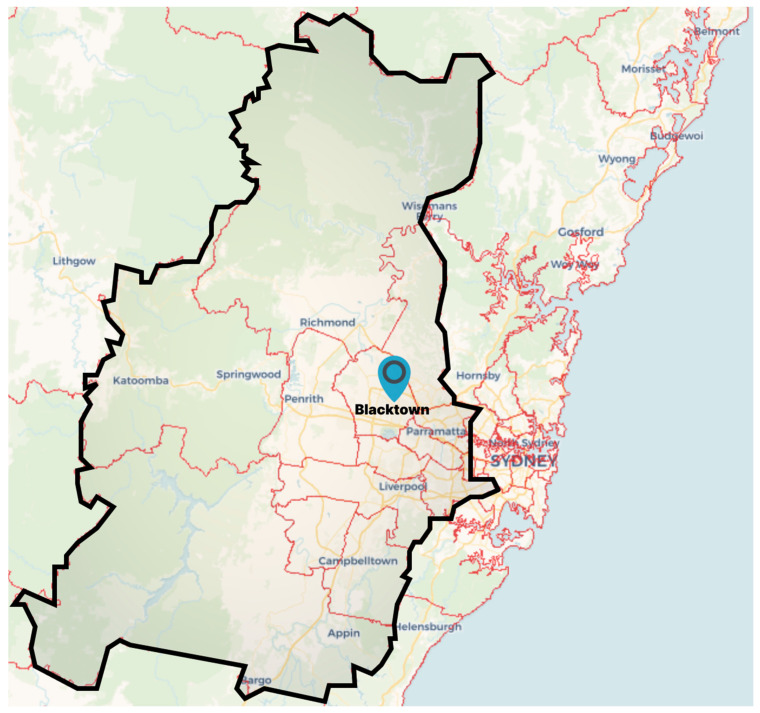
Map depicting Blacktown Local Government Area within Greater Western Sydney. National Map https://nationalmap.gov.au/about.html (accessed on 6 June 2022). Based on data supplied by: Australian Bureau of Statistics © Commonwealth of Australia CC-BY 4.0; © OpenStreetMap contributors ODbL; © CARTO CC-BY 3.0.

Greater Western Sydney (GWS) is a region in the western part of the Sydney metropolitan area, in the state of New South Wales (NSW). As of 2020, the region is home to ~2.5 million people. GWS is a rapidly developing, culturally diverse, and peri-urban region that is increasing becoming an area of national importance [32]. It has the third largest economy and one of the fastest growing populations in Australia. With a land area of 8982 square kilometer, GWS is subdivided into 13 local government areas (LGA). Blacktown, whose traditional custodian is the Darug Aboriginal people [33], is the LGA with the largest population in the region [34] and one of the largest urban Aboriginal populations in NSW [33]. Figure 1 depicts the location of Blacktown within GWS. Blacktown is located at the junction of two of the ‘three cities’ identified for major transformation in the Greater Sydney Region’s growth plan: ‘A Metropolis of Three Cities’ [35].

Blacktown is home to the GWS office of an international Non-Government Organisation (NGO). The NGO works in communities across Australia to improve the lives of underserved populations. The Blacktown office hosts cultural learning, strengthening, and connection programs for Aboriginal people, such as Elder’s groups, men’s groups, and women’s groups. This research was conducted in partnership with the Aboriginal community at the Blacktown office of the NGO. The representative of this community for the purposes of this research is the co-ordinator for the Aboriginal Elder’s Group. Three of the authors (KC, RI, AS) have worked with the co-ordinator on prior research, and they, along with one other author (JS), have extensive experience working with Aboriginal communities. Two other authors (TM, EDL) have prior experience conducting research in GWS.

GWS was particularly affected by COVID-19 during the third wave of the pandemic, which began in Australia in June 2021 with the spread of the Delta strain [36]. From the end of July until the middle of September 2021, several LGAs in GWS, including Blacktown, were subject to restrictions that limited residents’ movements to 5 km within their home [37,38]. The COVID-19 pandemic and these events in particular form a backdrop to this research, affecting data collection processes and the findings that emerged.

### 2.2. Sample

Typical of phenomenological studies [29], which have a small number of participants, ten people participated in this study. Seven participants were women. While participants were not asked about their health status, several volunteered such information in reference to their experiences of leaving home and using transportation. Of the ten participants, one mentioned having a disability, four mentioned using a walker, two mentioned being sick, two mentioned experiencing depression, and two mentioned being in pain. Three participants were interviewed twice, resulting in a total of 13 interviews. Participants are referred to using pseudonyms.

Participants were identified following a purposive sampling process, in which community-dwelling Aboriginal people aged 50 or over living in Greater Sydney who leave home at least once a week and who do not have regular access to a vehicle were invited to participate. They were identified and approached by the co-ordinator for the Aboriginal Elder’s Group at the NGO’s Blacktown office, an Aboriginal woman. As former or current members of the Elder’s Group, participants had an established relationship with the co-ordinator. Telephone interviews were conducted between May and November 2021 by two authors (AS, JS), both Aboriginal men with whom participants had no prior knowledge of or relationship with.

### 2.3. Data Collection

Participants were asked open-ended questions about the following topics: their participation in out-of-home activities, impacts of COVID-19 on their activity participation, transportation used to access activities, their experiences of transportation including what’s easy and what’s difficult, and ways they manage their activity participation and need for/use of transportation. While an interview guide was drafted prior to data collection, the pace, direction, and tone of interviews were set by participants. Interviews drew from yarning techniques [39]. Yarning is an Indigenous form of conversation that builds on the oral tradition of handing down information [40]. It is a culturally safe and respectful manner of inquiry that is recognised by Aboriginal people as an opportunity to share information through the telling of stories [40,41,42,43]. Yarning has its own convention and style [41,44]; it involves handing over control of the process to participants [44] and can be circular, meandering, and emergent [40].

The interviews, lasting approximately 30 min each, drew on three different types of yarning: social yarning, akin to rapport building; topic yarning, described as ‘a yarn with a purpose’ and, in a research setting, is akin to interviewing; and collaborative yarning, which involves discussions between the interviewer and the participant [40]. Thus, while an interview guide was prepared, its purpose was more to set the intention for the interviews than to direct the conduct of the interviews. Verbal consent was obtained before interviews commenced and audio recorded. Interviews were audio recorded and professionally transcribed, and transcripts were mailed to participants for verification. Participants received a $50 supermarket voucher to thank them for their time.

### 2.4. Data Analysis

Qualitative data analysis was guided by two analytic processes: decontextualisation and recontextualisation [29]. Decontextualisation involved breaking data, in the form of de-identified interview transcripts, into discrete parts and labelling them with codes. Recontextualisation involved drawing connections between codes, organising them into categories and/or hierarchies, and relabelling them to better reflect their specific contents. Two types of codes were defined, which were not mutually exclusive: answers to questions and emergent themes. Codes relating to questions in the interview guide were considered ‘answers to questions’. Codes addressing topics not in the interview guide were considered ‘emergent themes’. For example, a story about negative treatment from others was both an ‘answer to question’ because it relates to difficulties encountered, a topic on the interview guide, and an ‘emergent theme’ because it spoke to the type/quality of social interaction experienced, which was not a topic on the interview guide. Defining these two types of codes enabled us to follow an inductive approach, while also keeping our intentions for the interviews top-of-mind. Memos were used to summarise and explore insights and interpretations of the data as well. NVivo 12 was used to manage the data and aid analysis. Analysis was conducted by two authors, an Aboriginal researcher (KC) and a non-Aboriginal researcher (TM). While TM was responsible for carrying out the mechanics of data analysis (e.g., coding in NVivo), analytic decisions and interpretations were made collaboratively through fortnightly discussions and sharing of written materials such as memos and annotations.

## 3. Results

### 3.1. The Need for Transport

Participants engaged in various out-of-home activities. The local NGO was a routinely visited destination, hosting the Elder’s groups, men’s groups, and women’s groups that participants regularly attended. Participants also volunteered in their communities and engaged in leisure activities such as dining out, pursuing hobbies (e.g., making cultural artefacts), and participating in games (e.g., draws and raffles). Participants also visited family members, provided care for family members, and enjoyed recreation with family members such as watching grandchildren play sports. Other activities include shopping for everyday essentials and attending medical appointments. Underpinning these activities are three key values that drive participants’ need for transport: family, community, and culture.

#### 3.1.1. Time Together with Family

Spending time with family is highly valued by participants. Paisley, referring to visits with family, said she ‘lives’ to see her grandchildren. Ada goes shopping with her son before his afternoon shift at work, a routine they have been doing ‘for years, every Thursday’.

When participants reflected on COVID-19, the impact on kinship was always the first to be mentioned. Simone commented that she used to go to her son’s place ‘all the time’ but is not able to anymore. She talked about her 17 grandchildren and is saddened by the fact that restrictions on seeing family are ‘disconnecting us from everyone.’ Similarly, referring to COVID-19, Dora noted:

It’s really affected me ‘cause I’m really close to my family. That’s really affected me, not seeing them or having contact with them… You know, they can ring me and all that, but just not all being together and seeing one another. And hugging the grandkids (laughs). Yeah, I’ve really missed that.

While participants faced challenges navigating the transportation system, carrying out kinship priorities made the challenges more tolerable, and, at times, worth it. Rowena explained:

For me to get to [my daughter’s] place, it’s, like, three buses. And she rang me up and told me on Saturday that my grandson’s making his confirmation. And she can’t come pick me up ‘cause she’s got so much to do to get the three boys ready for church and get her husband organised. And she went ‘You’re gonna have to make your own way there, mum. I’m sorry.’ And I said ‘That’s okay’ because I didn’t wanna miss it, you know. It’s a special moment. I said to her ‘I’ll be there, don’t worry about me, I’ll get there.’ *Things like that I don’t mind*.

[emphasis added]

#### 3.1.2. Being a Part of Community

Participants voiced a desire to go to places where they can be part of community. Speaking of her Elder’s Group, Simone described it as the ‘main thing’ that she does, giving it a central role in her day-to-day life. She looks forward to it every Tuesday and said of the co-ordinators:

They make you feel so loved… I look at [them] as my sisters. Now, that’s what they’ve done to me. I mean [look] how much they’ve given me. They’ve given me my love back and strength back to be able to get out the front door and do something… It’s important to us, that group. It’s incredible.

The value that participants place on community was further emphasised when they reflected on the impact that COVID-19 had on their ability to maintain social connections. Rowena explained:

My doctor told me to go into self-isolation in March last year. And it’s just been so hard. Because I can’t see anyone. So, I’ve been here by myself. So, you know, I’d sit here and talk to the cat until he gets sick of me talking and puts himself to sleep in the wardrobe. So, it was, um, very stressful and very depressing. And, you know, I had some not good thoughts and feelings when I was just isolated for that long… It was a horrible time.

Being a part of community was such a priority to participants that, at times, it compelled them to endure the challenges associated with *getting* to those places of community in the first instance. Although she had to commute 2–3 hours each way via multiple bus and train trips to volunteer in a community engagement role, Rowena remarked: ‘I didn’t mind doing it because I loved ringing up the Elders’. Interestingly, being in community appeared to be both a value and an obligation, as suggested by Rowena’s comment:

When COVID hit, it was a bit of a relief, ‘cause I didn’t *have* to [emphasis added] get up and do that four hours of traveling every day. But, you know, since COVID and everything, we’ve been doing it at home, so, you know, that’s all good.

#### 3.1.3. Connecting with Culture

Participants’ out-of-home activities also represent opportunities to connect with their family heritage and culture. Simone told a story of coming together with her brother and cousins, on Country, visiting grave sites of her great-great-great-grandparents and finally putting together the puzzle pieces on her family history. Eric discussed his participation in a men’s group and how it was an opportunity to spend time with his ‘uncles and make [cultural] artefacts.’ Not being able to go out and pursue these activities negatively affected participants’ cultural well-being. Referring to COVID-19 restrictions on movement, Eric noted:

It was hard in the cultural way, yeah. With me being back on Country and you know, we’re used to having people around, you know, and people weren’t allowed to come and see you or, whatever like that. That was a very bad thing.

The sense of unease that some participants felt from COVID-19 stemmed not only from limitations on their ability to connect with culture, but also from concerns about the safety of their community, as individuals *and* as keepers and stewards of Aboriginal culture. Simone explained:

What’s [COVID] done to me, I’m frightened now. I’m frightened to go out. I fear, you know, a lot of those Elders getting out there, uh, ‘cause that’s the thing that’s bothering me, too, ‘cause we’re losing Elders… It’s every one of the Indigenous mob, you know? It’s hitting all of our people. Where is our culture going?

### 3.2. The Use of Transport

Participants utilised multiple modes of transportation to access their out-of-home activities including walking—for short trips—train, light rail, and bus. Participants who met eligibility criteria and qualified through an assessment also used transport services funded by the Australian Government’s Aged Care Scheme and National Disability Insurance Scheme (NDIS) respectively. Through these schemes, eligible and qualified participants are allocated a specific amount of funded transport services and must arrange these services themselves, for any destination of their choosing, through private service providers. These include community transport organisations, for transport on community transport buses, and home care organisations, for transport on personal vehicles driven by individual carers who provide assistance with everyday living. Participants also used transport organised by individual services, like the local NGO, who sometimes transport participants to and from their location where programs and services are held. Lastly, participants arranged transport through informal social arrangements. Participants get driven by family members and friends and carpool with peers going to the same destination. When using transport, participants had to negotiate several barriers, including the rules and procedures of the transport system, temporal complexity and uncertainty, stigma, and condescension.

#### 3.2.1. Rules and Procedures of the Transport System

While the physical design of public transport systems may include accommodations for people with varying mobility levels, the service design in terms of the rules and norms governing the user experience may not. Several participants voiced that they struggle to navigate public transport with a walker and gave examples of having to lift the walker on and off buses as well as maneuverer it to and from lifts and platforms at train stations. One way that participants have had to negotiate the rules and procedures of the transport system pertain to physical accessibility, as explained by Ivan:

When I started using a walker, … I rang up Transport for New South Wales (Transport for New South Wales is the publicly funded state transport agency), [and] I asked them about, uh, the ramp...the thing you use for the wheelchairs. And they said ‘Anyone who has got a walking aid, even a walking stick, is allowed to use a ramp.’ So I go out and, on the next bus, asking, ‘Can I use the ramp, mate?’ He says ‘No, that’s only for wheelchairs.’ I said ‘Um, well, Transport for New South Wales said I can use it.’ ‘No, no, no, no. Sorry, you can’t use it. No, no.’ And that, that really got me, you know? I would ring Transport for New South Wales [again] and they said ‘They’re supposed to let you use it. Take [down] the driver’s number, the bus number, the route, and what time and ring us back and tell us, and we’ll tell the bus driver.’ And this kept on going for about 12 months. And like every year, I said ‘Why don’t you tell all the bus drivers to do the same for all disability people?’ ‘Oh, we can’t do that. Only once you complain about [it].’ You know? And I’m thinking, this is ridiculous. Oh, it was getting really frustrating, yeah.

Another set of rules and procedures standing between participants and their use of transport involves the need to schedule community transport in advance. While participants preferred community transport for its convenience at the time of travel, it comes with a major inconvenience prior to travel. Participants expressed that the need to book community transport in advance, which, while understandable from a service provider perspective, is not compatible with the reality of needing to travel spontaneously. Rowena explained:

You’ve gotta give them, like, three days’ notice to get transport. And sometimes if you’re sick, you need to go there that day. If you’re sick and you don’t have money for a taxi, you know, it just makes it hard. [Community transport providers] do their best, but if you ask here it is a problem.

For those with no other transport option, being unable to travel by community transport on short notice means that they are not able to attend their out-of-home activity commitments. For those with other transport options, they have had to resort to a less desirable option such as taking the bus, which comes with temporal complexity and uncertainty (see next subsection). While the ‘hassle’ of public transport compared to community transport and its status as the second-choice option to community transport were emphasised throughout the interviews, one participant found the need to book in advance so challenging that public transport seemed easy in comparison. Eric contrasted the inconvenience of needing to book community transport in advance with the ease of deciding to take the public transport and doing so in that moment:

[For community transport,] it’s pretty hard because you’ve got to ring a couple of days before so you can book. [That] part is really hard, but the easy part is, like I said, I just catch a train and go out to the west side.

The rules and procedures of the broader social care system can also impede participants’ ability to travel to valued activities or destinations. When asked if he had transport support through the NDIS, one participant provided insight into the process of qualifying for NDIS benefits. In this participant’s situation, he was repeatedly turned down for benefits, and when further prompted, revealed that he was unsure about how to advocate for his needs, not knowing what supports and services were available. Addressing the question of transport support through the NDIS, Eric responded:

That’s a laugh. A couple years ago, they were saying if I don’t register I’m going to lose my benefits. That’s what the letter said. I think I’ve still got it. And so I did that. I rang up, asked for the forms and everything, mailed them away, and got an email back saying, ‘Sorry, we can’t put you on.’ It was a joke. To me, it was a joke. I thought, ‘Well you’re kidding aren’t you?’ So I did the right thing and I got the doctor to sort everything out. And they said ‘Sorry but you don’t qualify’. And I thought ‘Well with all the problems I got, I shouldn’t have any problems getting on the NDIS’ but no, no they didn’t want me.

When the interviewer noted that Eric was able to request, in his care plan, cultural support in terms of travel assistance to his ancestral lands, Eric remarked:

Oh I didn’t know they do that. I thought it’d be going a bit overboard asking for [that]. [Especially since] they didn’t want to do anything for me… That is one thing I would love to do. It’s something I’ve never done before. And it’s only been the past 10, 15 years that I’ve actually known about my Aboriginal side and I’m wanting to know more.

This participant’s experience suggests that challenges navigating the rules and procedures of broader social care systems can hinder one’s ability to pursue meaningful life activities.

#### 3.2.2. Temporal Complexity and Uncertainty

When describing their experiences with public transport, participants commented on the burden that it places on their time. Aside from having to wait for the bus due to infrequent or irregular bus schedules, participants described their need to co-ordinate multiple bus and train times. Rowena provided an example:

When I used to go into the office at Blacktown [to volunteer for the local NGO], I used to catch a bus at, um, 7:30 in the morning from my place to, um, Chester Hill Station. Then I’d get off that bus, stay at the same bus stop, and seven minutes later, another bus came, and I used to get that to Parramatta. And then I’d get off at Parramatta and catch a train from Parramatta to Blacktown…It’d take me two hours in the morning, and then sometimes it’d take me two and a half hours maybe three coming home.

Not only is public transport associated with significant time demands and complexity in planning, but it also compels participants to tolerate a level of uncertainty with their time. Rowena described traveling to visit friends and family but not being able to stay long due to the need to adhere to public transport timetables. She would sometimes need to take a taxi to her destination because she did not know when she would have to leave home if taking the bus.

All my friends, like, live in different suburbs. So, if I wanna go and visit them, sometimes it’s, like, four buses in the day, and you can’t stay long, because you’re trying to work out the timetable. So, it’s really hard to catch up with people… For me to get to [my daughter’s] place, it’s, like, three buses. And she rang me up and told me on Saturday that my grandson’s making his confirmation... So, I’m gonna have to catch a taxi to go there, because, you know, I don’t know what, what time I’d have to leave home to get to the church on time. And I [would] have to catch a couple of buses.

This temporal complexity and uncertainty is shaping and restricting the way participants are able to live their lives and fulfil kinship obligations and preferences. Rowena summarised the impact of the lack of bus service on Sundays and public holidays succinctly, stating that, as a result ‘you’re stuck in the house and you can’t go visit anybody anyway.’

#### 3.2.3. Stigma

Participants provided examples of the stigma that they have had to contend with when engaging in out-of-home activities. This experience relates to, but goes far beyond, the use of transportation. Ada recounted her experience, relating it to the colour of her skin. She started by referring to a dentist appointment that she went to via train:

But when I get on the train or the bus, I get this feeling that ‘Should I be on there or not?’ It’s just one of those feelings you know? Even on the planes too. Doesn’t matter where you go, dear, you get that funny feeling that, ‘Oh, do I belong there or not?’

When reflecting on her experience of being stigmatised, Ada first thought of the various transport modes that she has used. But then, she recalled similar experiences in other public places:

It’s not only public transport, it’s everywhere. That we’ve got that stigma of we shouldn’t be there… I could be going out for lunch, you know and you can feel it when you walk in the doors. Um, the pools. I go [to] activities in the pools, and they stare. Going to, um, even shopping… I could walk out of my own [apartment] unit and [they’d] say ‘Oh what’s she doin’ comin’ out of there?’ Going into, you know, important places where the doctor sends ya or like hospitals… Once we get in that door and into the corridor, boom. Eyes. Just stare at ya. And they must think ‘Oh what is she doin’ here.’ But there’s all nationalities you know, in one place. So why did they pick us? To say ‘Youse don’t belong here.’ But they’re on our land, aren’t they?

The feeling that she referred to has led Ada to question her own right to be in public. She continued:

Then you think, ‘Are we entitled to be there?’… Like, you think, ‘Oh, should we get a Koorie bus for our people, or a Koorie train for our people?’… Like I’ve been to a lot of, um, restaurants [and think] ‘Should we just eat fast and get out?’… Do we have to have a white person to come along, you know, just [for them] to say, ‘Oh she’s with her or him. That, that must be okay.’ You know.

#### 3.2.4. Condescension

Participants also discussed the treatment they received by others when interacting with the transportation system. While the experience of being stigmatised is more about an *attitude* of disapproval and discrimination directed at them, here, participants are describing the *behaviour* of others toward them. Continuing his earlier account about negotiating with bus drivers to use the ramp, Ivan described the way they treated him, refusing to let down the ramp:

Someone would say ‘What? All those old ladies [are] getting up here [on the bus]. Can’t you get up?’ All this type of thing. They abuse you. At least one person said to me, ‘What do you think you are? The king?’ (laughs). These bus drivers are famous here eh? Treating [you] like you’re rubbish.

The condescending treatment that Ivan received from bus drivers was directed as his mobility impairment and need for a walker. He continued:

And even going down to the shop, the woman [who] served me was treating me like, uh, like a little kid. Like, people, what I’ve noticed, is that... a lot of people treat disability people, uh, as though... if they’ve got a physical disability, there’s also a mental disability there. The way they talk to you.

In these interactions, Ivan was treated by service professionals as though they were superior, reducing him to someone ‘like a little kid’ or ‘like rubbish’. Other participants shared experiences of negative treatment by others, where they were ignored and rendered invisible. In Paul’s account, his presence was not acknowledged, and his personal space was encroached upon. In Rowena’s account, her need for assistance was not responded to.

Cause I’ve got arthritis and that, I’ve got to use a lift [to access the trains]. And sometimes it’s hard when you get people pushing into the lift and that. Even younger people, you know, and they’re not even looking up, might have a knee go on me.

(Paul)

Public transport is sometimes difficult for me because I use a walker... and trying to lift the walker on and off the bus [is hard] because people aren’t very helpful in these days.

(Rowena)

## 4. Discussion

This study explored how older Aboriginal people in Greater Western Sydney experience the transportation system, providing an Indigenous-centred view of the accessibility of transportation options in society. Through interviews with community-dwelling older Aboriginal people, we identified several barriers that older Aboriginal people had to contend with when living out their family, community, and cultural participation values.

In addition to the cognitive labour required to decipher the rules of the transport system and organise commitments to match the scheduling of transport services, older Aboriginal people in this study experienced stigmatising attitudes and condescending treatment from service professionals and the public when traveling. These barriers rob older Aboriginal people of the ability to travel when and where needed with ease, which diminishes their capacity to pursue valued life activities centred on family, community, and cultural participation. This diminished capacity for family, community, and cultural activities is incompatible with the social determinants of Indigenous health, which identify the ability to attain one’s own aspirations as integral to good health [45,46]. Congruent with the Aboriginal and Torres Strait Islander definition of health, when individuals, such as the older Aboriginal people in this study, are not able to fulfil their aspirations, the well-being of the whole community is compromised [47].

The barriers posed by the rules and procedures of the transport system and the temporal complexity and uncertainty associated with using transport bring into question whether there exists a gap in existing service design practice. Service design, as the activity of planning and organising all components of a service to improve customer experience and the quality of the service encounter, requires an appreciation for the realities of end-users [48,49]. This study brings to light a reality for older Aboriginal people that is based on fragmented communication between bus drivers and the state transport agency, affecting physical access onto buses, lengthy lead time for community transport bookings, affecting the ability to travel spontaneously or urgently, unclear qualifying criteria for transport support through the broader social care system, affecting the ability to receive essential transport assistance, and complex public transport timetables, affecting the ability to pursue activities of value. Addressing these realities require *valuing* older Aboriginal people’s perspectives and experiences and *including* them in generating ideas for solutions. While these principles are core to service design practice [48,49], this study raises the possibility that older Aboriginal people are overlooked as a user segment. Indeed, transport services are largely designed to suit the working population, with less consideration for older people [50]. Furthermore, given observations that the interests of Aboriginal people are ignored in policy implementation [20], it may be that older Aboriginal people are not considered in the design of the transport system.

The experiences of stigma and condescension point to the racism that pervades the lives of Aboriginal people—alluding directly to Indigenous scholar Margaret Kovach’s statement that ‘the relationship with the settler society impacts our world daily’ [24]. While racism is systemic, existing on a broad societal level, the experiences of racism by the older Aboriginal people in this study, in relation to their use of transport, raises several possible implications for the transportation system. Specifically, it questions whether the diversity and inclusion agenda goes far enough, with the example of bus drivers’ condescending behaviour suggesting a potential need for cultural awareness training. It also suggests a potential need to consider the social determinants of Indigenous health, which recognise the adverse health effects of racism [45,46,51], when conceptualising and designing a transportation system that maximises transport-related benefits and minimises transport-related burdens [52].

Given the rapid growth of GWS, and its large urban Aboriginal population, it is imperative that the current trajectory that underserves older Aboriginal people be disrupted, lest exacerbating existing patterns of exclusion and inequity. With the progress of the age-friendly city agenda [53], city leaders have an increasing obligation to shape cities so that they are physically accessible and socially inclusive for older people from all walks of life. This requires designing for diversity and reducing inequities in older people’s access to goods, services, opportunities, and social life [54]. While this study is specific to GWS, the findings may have relevance in other contexts, which is an avenue for further exploration.

By incorporating yarning techniques in the data collection process, this study invited participants to engage in a familiar communication process so that they can talk freely about their experiences [40,42] and be the authority of their knowledge [55]. This was evident in the tone of the interviews, as participants became discernably more open and absorbed in the conversation as it progressed. This demonstrated the trust that was unfolding and the relationship that was being built with the interviewer. This was also evident in the findings, as issues not set out in the interview guide emerged, allowing a more holistic exploration of older Aboriginal people’s experiences of the transportation system. Furthermore, yarning is congruent with an Indigenous axiology based on relational accountability [26], whereby yarns carry within them the shared lived experience of families and communities [43]. This is an important ethical point as yarning techniques allow for the development of culturally safe research.

Due to COVID-19 measures, interviews were conducted one-on-one via telephone, rather than face-to-face in a group setting, which would have been a more familiar and relaxed setting and might have led to more in-depth discussion. As interviews occurred during COVID-19 restrictions on movement, participants’ sharing of information about their out-of-home activities and use of transportation might have been more reflective and recall-based than otherwise. Another methodological observation is that both Aboriginal interviewers are male. Due to the gendered roles that Aboriginal people assume as they age and generational knowledge being transmitted along gender lines [9], this might have influenced the nature and/or depth of the conversations with female participants. Although the first author (TM) identifies as a non-Indigenous person, the close collaboration between the first and senior author (KC), an Aboriginal person, and the discussions with both Aboriginal interviewers during the data collection and analysis stage ensured the research remained Indigenous-centred.

The findings of this study question conventional discourse that holds Aboriginal people responsible for not fitting in with dominant norms and institutions [20]. Rather, this study questions whether the opposite might be true—that transport decision-making did not adequately engage with older Aboriginal people as a user segment in service design, the diversity and inclusion agenda, nor the social determinants of Indigenous health in envisaging a socially sustainable transport system.

## 5. Conclusions

Several barriers diminish older Aboriginal people’s capacity to travel when and where needed with ease, impeding their ability to pursue valued life activities centred on family, community, and cultural participation. By bringing to light these structural and interpersonal barriers, this study provides an Indigenous-centred view of the accessibility of transportation options in society. Furthermore, this study suggests three potential ways that the current trajectory that underserves older Aboriginal people could be disrupted, relating to service design, the diversity and inclusion agenda, and the social determinants of Indigenous health.

## Data Availability

The data generated and analysed during the current study are not publicly available because study participants did not consent to the data being made available to other researchers.
